# Increasing ibuprofen degradation in constructed wetlands by bioaugmentation with gravel containing biofilms of an ibuprofen‐degrading *Sphingobium yanoikuyae*

**DOI:** 10.1002/elsc.201900097

**Published:** 2020-01-13

**Authors:** Eduardo Marcos Balciunas, Uwe Kappelmeyer, Hauke Harms, Hermann J. Heipieper

**Affiliations:** ^1^ Department of Environmental Biotechnology Helmholtz Centre for Environmental Research ‐ UFZ Leipzig Germany; ^2^ Department of Environmental Microbiology Helmholtz Centre for Environmental Research ‐ UFZ Leipzig Germany

**Keywords:** constructed wetlands, ibuprofen, *Juncus effusus*, *Phalaris arundinacea*, Sphingobium yanoikuyae

## Abstract

The aim of this study was to investigate the removal of ibuprofen in laboratory scale constructed wetlands. Four (planted and unplanted) laboratory‐scale horizontal subsurface flow constructed wetlands were supplemented with ibuprofen in order to elucidate (i) the role of plants on ibuprofen removal and (ii) to evaluate the removal performance of a bioaugmented lab scale wetland. The planted systems showed higher ibuprofen removal efficiency than an unplanted one. The system planted with *Juncus effusus* was found to have a higher removal rate than the system planted with *Phalaris arundinacea*. The highest removal rate of ibuprofen was found after inoculation of gravel previously loaded with a newly isolated ibuprofen‐degrading bacterium identified as *Sphingobium yanoikuyae*. This experiment showed that more than 80 days of CW community adaptation for ibuprofen treatment could be superseded by bioaugmentation with this bacterial isolate.

**Abbreviations**
CW: constructed wetlands
CW‐B: constructed wetland planted with *Juncus effuses* inoculated with bioaugmented gravel
CW‐J: constructed wetlands planted with *Juncus effusus*
CW‐P: constructed wetland planted with *Phalaris arundinacea*
CW‐U: constructed wetland unplanted constructed wetlands

## 1 INTRODUCTION

The widespread occurrence of pharmaceutical compounds in the aquatic environment has been considered as an emerging environmental issue for many years 1, particularly as significant amounts of pharmaceutical residues are suspected to enter rivers, streams, and surface waters through the effluents of wastewater treatment plants 2. In this regard, alternative approaches and strategies for the elimination of micropollutants are a matter of great interest. Recently, several studies have shown that constructed wetlands have great potential for the removal of pharmaceutically active compounds 3, 4, 5, 6. Constructed wetlands (CWs) are already widely used for decentralized wastewater treatment due to the low operational and maintenance costs as well as low energy input 5.

Several pathways for the biodegradation of organic pollutants may occur under different redox conditions in CWs systems. Processes such as metabolic transformation, contaminant accumulation, plant uptake, and phytovolatilization may be relevant for some organic chemicals 6. The relative importance of a process shifts significantly depending on the organic contaminant being treated, the wetland type (e.g. subsurface‐flow wetlands or surface‐flow wetlands, horizontal flow or vertical flow) and operational design (e.g. retention time), the environmental conditions, the type of vegetation within the system, as well as the soil matrix 6.

In wetland systems, the intrinsic physical, chemical, and biological processes may occur simultaneously for water quality improvement, including volatilization, sorption and sedimentation, phytodegradation, and plant uptake 7. Complex microbial communities are shaped by the interplay of microorganisms, water content, soil, and plants 8. They play a key role in wetland biogeochemical cycles and for contaminant degradation in constructed wetland systems 9, 10. The presence of specific microorganisms can be the dominant factor for pollutant removal 9, 11.

Ibuprofen, a non‐steroidal anti‐inflammatory drug, was selected for this study because of its widespread usage and persistent occurrence in wastewater. Verlicchi and Zambello 12 reviewed 118 pharmaceutical compounds which have been frequently detected in raw urban wastewater and indicated that ibuprofen was considered as the most commonly inquired anti‐inflammatory drug with the highest influent concentration of 373 µg/L in raw urban wastewater.

Investigations into ibuprofen environmental impacts have found induced changes on growth and predominance of algae and duckweed, the timing of spawning by medaka (Japanese rice fish *Oryzias latipes)*, riverine biofilm communities at relevant concentrations, as well as changes in microbial diversity in aquatic mesocosms 13. Ibuprofen was also reported to alter human testicular physiology, altering the endocrine system via selective transcriptional repression, thereby inducing compensated hypogonadism 14.

Although various removal mechanisms may contribute to the total elimination of pharmaceutical compounds in constructed wetlands 10, for polar acidic pharmaceutical compounds like ibuprofen, microbial degradation is considered to be most significant 15.

It usually takes two months for wetland plants and associated microbes to adapt to and propagate in newly constructed wetlands 16. In this study, we aimed at superseding this phase by bioaugmentation of a CW to achieve fast ibuprofen elimination. Gravel previously loaded with a bacterial isolate um capable of utilizing ibuprofen as a sole carbon and energy source was inoculated into a system without prior contact with ibuprofen. Finally, we report the importance of plants in CWs, a comparison between two typical CW plants in relation to ibuprofen removal, as also the increased performance of a bioaugmented CW in comparison with a non‐bioaugmented CW. To the best of the authors’ knowledge, this is the first time that *Sphingobium yanoikuyae* was isolated from a municipal wastewater large‐scale CW and successfully inoculated in a laboratory‐scale CW.

## 2 MATERIALS AND METHODS

### 2.1 Experimental design and conditions

The simulation of horizontal subsurface flow constructed wetlands conditions and carbon cycling related to rhizosphere processes was conducted in four laboratory‐scale constructed wetlands. Constructed wetlands planted with *Juncus effusus (*CW‐J) was planted with *Juncus effusus*, while constructed wetland planted with *Phalaris arundinacea (*CW‐P) was planted with *Phalaris arundinacea* and constructed wetland unplanted constructed wetlands (CW‐U) was left unplanted. For the second phase of the experiment, a constructed wetland planted with *J. effusus* (CW‐B) was used to test the effect of bioaugmented gravel. The CW models were 1 m long × 0.15 m wide × 0.35 m deep, resulting in a surface of 0.15 m². Measurements were conducted from April 2016 to March 2017. Each CW was filled with fine gravel of a sum mass amount of 54 kg, an average particle size of 2–4 mm and a porosity of 38.3%.

**PRACTICAL APPLICATION**

We describe the successful application of bioaugmentation of a constructed wetland by inoculation of gravel loaded with biofilms of a newly isolated ibuprofen‐degrading bacterium identified as *Sphingobium yanoikuyae*. Constructed wetlands that had been bioaugmented in this way showed an immediate degradation of the compound, whereas regular constructed wetlands deserved a more than 80 days of community adaptation period.

The CWs were established in a greenhouse with controlled environmental conditions, representing an average summer day in a moderate climate. The temperature was set at 22°C from 6 a.m. to 9 p.m. and at 16°C overnight. An artificial light source (Master SON‐PIA 400W, Philips, Belgium) was set to turn on when the natural light fell below 60 klx during daytime.

The water level within the CWs was maintained at 1 cm below the gravel surface by the use of a syphon type of outflow system. Before the measurements and ibuprofen inflow started, all four CWs had six month acclimatization period within the greenhouse. During this period, each CW was continuously fed with artificial wastewater, at an inflow rate of 6 L/d; corresponding to a hydraulic loading rate of 40 L/m²/d, with a theoretical hydraulic retention time of 2.5 days.

The artificial wastewater was prepared in accordance with standard artificial wastewater protocols (DIN‐38412‐T24, 1981). However, the sulfate load was reduced to limit microbial dissimilatory sulfate reduction processes. The artificial wastewater contained (in mg L^−1^): 79.1 NH_4_Cl, 24.7 K_2_HPO_4_, 4.7 NaCl, 2.7 CaCl_2_ ⋅2 H_2_O, 1.1 MgCl_2_⋅6 H_2_O, 0.6 Na_2_SO_4_ (dissolved in deionized water). Moreover, the artificial wastewater contained 1 ml L^−1^ of trace mineral solution 17 comprising (in g L^−1^): 1.0 NaEDTA, 1.0 FeSO4⋅7 H_2_O, 0.8 MnCl_2_⋅4 H_2_O, 1.7 CoCl_2_⋅5 H_2_O, 0.7 CaCl_2_⋅6 H_2_O, 1.0 ZnCl_2_, 1.5 CuCl_2_⋅5 H_2_O, 0.3 NiCl_2_⋅6 H_2_O, 0.1 H_3_BO_3_, 0.1 Na_2_MoO_4_⋅2 H_2_O, and 0.02 Na_2_SeO_3_⋅5 H_2_O. The resulting inflow composition (in mg L^−1^) was: 20.7 ammonium‐N, 4.4 phosphate‐P, 0.2 sulfate‐S and 36.0 inorganic carbon. After the acclimatization period the artificial wastewater was supplemented with 50 mg L^−1^ Ibuprofen sodium salt (Sigma‐Aldrich) as the only externally added organic carbon source.

### 2.2 Isolation of ibuprofen‐degrading bacteria

In order to isolate ibuprofen‐degrading bacteria, samples were taken from an operational large‐scale CW in Langenreichenbach, Saxony, Germany. The sampled site was a pilot‐scale horizontal flow CW planted with *Phragmites australis*. The system is 4.7 m long, 1.2 m wide and has a saturated water depth of 50 cm. The system was dosed with pre‐treated municipal wastewater at a rate of 5 L every 30 min, leading to an average inflow rate of 0.18 m^3^ d^−1^. The system is in operation since 2010 under changing climatic and organic loading conditions as described by Carranza‐Dias 2014 18. The experimental site is described in detail by Nivala et al. 19. Previous measurements in Langenreichenbach found inflow concentrations of ibuprofen between 11 and 92 µg L^−1^ 18.

20% v/v of the sample was added to mineral salt medium enriched with 500 mg/liter ibuprofen as described by Murdoch and Hay 20. This procedure led to the isolation of a bacterium capable to use ibuprofen as sole source of carbon and energy. A single colony was isolated and identified by PCR amplification and sequencing of the 16S rRNA gene fragment, using the universal primer pair 27F (AGAGTTTGATCMTGGCTCAG) and 1492R (TACCTTGTTACGACTT) 21. The bacteria were identified as a *Sphingobium yanoikuyae* (100%) strain.

### 2.3 Loading of gravel with *Sphingobium yanoikuyae* for bioaugmentation of the CW

2.5 kilos (5% w/w) of CW gravel was added to 1‐L bottles, autoclaved and then treated with ibuprofen mineral salt medium, inoculated with *S. yanoikuyae* and incubated at 30°C in a vertical incubator at 250 rpm. The treatment lasted seven days, with medium renewal and inoculation every 24 h. After the treatment period, a biofilm was visible around each gravel stone. The bioaugmented gravel was spread all over the length of the CW‐B, below the water level. The addition of ibuprofen to the inoculated CW was started immediately after the inoculation of the bioaugmented gravel.

### 2.4 Analysis and calculations

All samples were harvested directly from the system outflow using a peristaltic pump (BVP Z, ISMATEC SA, Switzerland). The water was pumped through a flow cell, and the redox potential (SenTix ORP Pt/Ag+/AgCl/Cl‐, WTW, Germany), pH (pH 539, WTW, Germany) and temperature (Checktemp1, Hanna Instruments, Germany) were recorded. To measure the dissolved oxygen concentration, an optical trace oxygen mini sensor (FTC‐TOS7‐PSt3, PreSens GmbH, Germany) and a Fibox‐3‐Trace single channel fiber‐optic trace oxygen meter were used (PreSens GmbH, Germany).

Ibuprofen concentrations were analyzed in an HPLC prominence line (Shimadzu, Japan), equipped with LC Solution Postrun Analysis software, a degasser DGU‐20A3, a binary gradient pump LC‐20AB, an autosampler SIL‐20A, a column oven CTO‐20AC and a UV‐detection system SPD‐M20A. The column and security guard used were both LiChrospher 100 RP‐18 (5 µm) (Merck KGaA, Germany). The column was operated at a constant temperature of 30°C. The mobile phase consisted of 0.1% v/v formic acid in water (Eluent A), and methanol (Eluent B) and the gradient program was selected to be 20% B to 80% B (25 min).

## 3 RESULTS AND DISCUSSION

### 3.1 Overall ibuprofen removal efficiency

Plants in constructed wetlands play an important role for the removal of pharmaceutical compounds. They stimulate the development and activity of microbial communities which are supported by rhizodeposition products like exudates, mucigels, and dead cell material in the rhizosphere 22, 23, 24. Thus, the presence and the kind of plants in a constructed wetland are important criteria for the removal efficiency of pharmaceuticals in CWs. Matamoros et al. 25 found that the predominant removal process of ibuprofen was microbial degradation which was probably associated with biofilms on the surface of plant roots.

For the current study, *J. effusus* and *P. arundinacea* were selected as the test species since they are frequently used for the treatment of sewage in wetland systems in Europe and in North America 26.

**Figure 1:**
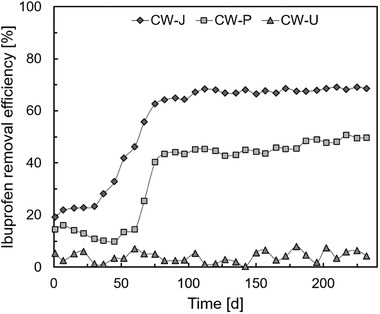
Ibuprofen removal efficiency of the constructed wetlands planted with *Juncus effusus* (CW‐J), *Phalaris arundinacea* (CW‐P) and an unplanted system (CW‐U)

Figure 1 shows the ibuprofen removal efficiencies for the system planted with CW‐J, *P. arundinacea* (CW‐P), and CW‐U tested during 232 days of sampling. Eighty days after the start of the experiment, both planted systems showed higher removal efficiency (64.2% for the CW‐J; 43.4% for the CW‐P) in comparison with the unplanted system (2.5% for the CW‐U). After 130 days, CW‐J and CW‐P showed ibuprofen average removal efficiencies of 68 and 46.9%, respectively, while CW‐U showed an average removal rate of 4.5% (Figure 1).

The higher ibuprofen removal efficiency in planted systems compared to the unplanted one can also be explained by the release of oxygen from the root zones of plants that might counterbalance the chemical and biological oxygen consumption by microorganisms in the rhizosphere. Many plant species are able to release oxygen around their roots in constructed wetlands 24, and different species release different rates of oxygen 27. The systems planted with *J. effusus* showed higher average oxygen concentrations (2.02 ± 0.31 µg L^−1^ for CW‐B and 1.95 ± 0.26 µg L^−1^ for CW‐J) than the system planted with *P. arundinacea* (0.94 ± 0.31 µg L^−1^) and the unplanted system (0.30 ± 0.09 µg L^−1^). The oxygen released in the rhizosphere can also sustain chemical oxidation of contaminants in wastewater, favor the development of aerobic microorganisms in the rhizosphere and induce more efficient biodegradation processes 23, 28.

It remains unclear, if the higher ibuprofen removal efficiency of *J. effusus* in comparison to *P. arundinacea* was due to higher plant absorption of ibuprofen or to higher oxygenation of the rhizosphere, as discussed before. Microbial degradation has repeatedly been reported to be the main pathway for ibuprofen removal, but plant uptake and metabolization may also play a role 5. Nevertheless, the higher removal efficiency of *J. effuses* in the initial experiments clearly point at its superiority to *P. arundinacea* for the inoculation experiment phase.

Ibuprofen removal efficiency is less affected by the plant species than by the presence or absence of plants in the present study (Figure 1). Other studies did either not find differences between different plant species for nutrient and organic matter removal 29, 30, 31, or pathogen removal 31, 32, or they reported higher removal for some selected plant species 32, 33, 34. In some other studies, differences existed but were not significant 35 or changed among the years of operation 36, 37. Also, unvegetated systems exhibited higher removal of clarithromycin and trimethoprim 3 than vegetated ones, presumably mainly due to more direct insolation and higher concentrations of Chlorophyta algae in the former.

High concentrations of pharmaceuticals are known to affect plant growth in terms of average biomass. Kotyza et al. 38 found yellowing and desiccation of shoots of *Phragmites australis*, when it was exposed to 0.2 mM (i.e. 41 mg L^−1^) of ibuprofen, a concentration similar to the present study (50 mg L^−1^). The effects of ibuprofen in both systems planted with *J. effusus* were also evident in our study, where the average stalk densities of the augmented and non‐augmented systems were reduced by 20% and 16%, respectively for each system after supplementation with ibuprofen (data not shown).

### 3.2 Effects of gravel bioaugmentation on ibuprofen removal in constructed wetlands

**Figure 2:**
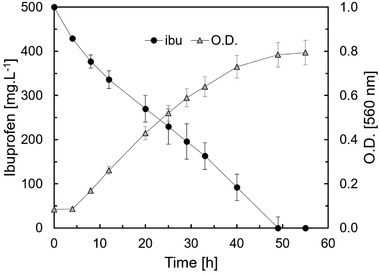
Ibuprofen concentration (●) and optical density (Δ) of *Sphingobium yanoikuyae* culture in mineral medium with ibuprofen as the sole carbon and energy source

For the second phase of the experiment, gravel previously loaded with biofilm of *S. yanoikuyae* was incorporated into a constructed wetland planted with *J. effusus* as described at Section 2.2. The bacteria were cultivated in a mineral medium with ibuprofen as sole source of carbon and energy (Figure 2). A yellow color appeared in the supernatant when *S. yanoikuyae* grew on liquid media containing ibuprofen. This yellow color faded upon acidification and reappeared upon neutralization. This phenomenon is an indication for the presence of the meta‐cleavage pathway of aromatic degradation catalyzed by these bacteria 20.

**Figure 3:**
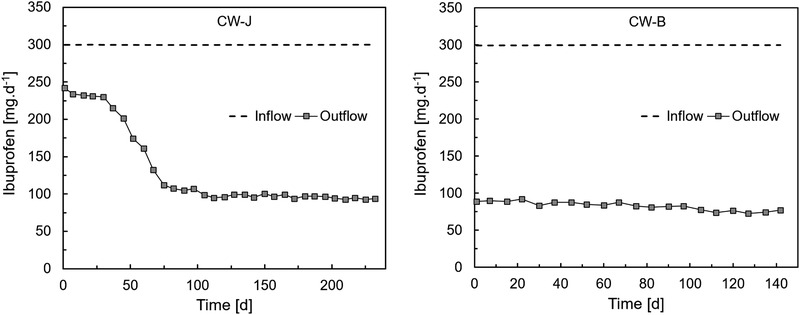
Ibuprofen concentration [mg/d] of an untreated constructed wetland planted with *Juncus effusus* (CW‐J) and one planted with *Juncus effusus* to which gravel loaded with biofilms of *Sphingobium yanoikuyae* were incorporated (CW‐B)

*Juncus effusus* was selected for the gravel inoculation experiment because of its positive influence on ibuprofen removal, as described above. Figure 3 shows the load of ibuprofen in the outflow and inflow, for the non‐bioaugmented (CW‐J) and bioaugmented (CW‐B) constructed wetlands during the 232 days of the experiment. The inoculation of bioaugmented gravel in CW‐B resulted in an immediate total removal rate of ibuprofen of 70%, and an average of 73% during the 142 days of measurement. The average removal rate in CW‐B was 5% higher than that of CW‐J measured during the period of highest performance, between 130 and 232 days of the experiment.

Water, root and gravel samples were collected from CW‐J, CW‐P and CW‐U and also from CW‐B before bioaugmentation. Sampling followed the same method described for bacteria isolation in Section 2.2. *S. yanoikuyae* was not found in any of the CWs suggesting that ibuprofen was being degraded by other species in the non‐bioaugmented systems.

### 3.3 Influence of the redox condition on the ibuprofen removal efficiency

Very different microbial carbon and nitrogen turnover processes occur simultaneously in CWs treating wastewater. The heterogeneity of the wetland rhizosphere allows different redox conditions controlled by and controlling aerobic and anaerobic microbial processes to exist simultaneously in the same system 27. The specific operating conditions of the CWs which are characterized usually by slow flow, macro‐gradients of concentrations, the existence of hypoxic areas, preferential flow and diurnal cycles makes the evaluation of rhizosphere redox processes difficult 39.

**Figure 4:**
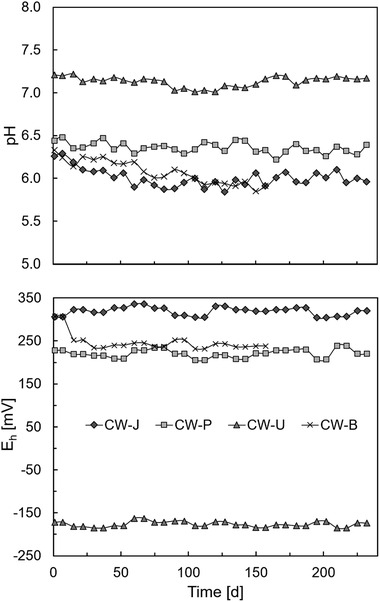
pH and redox potential (Eh) of the outflow on the wetland planted with *Juncus effusus*, *Phalaris arundinacea*, unplanted and with treated gravel during the 240 days of measurement

The average final effluent redox potentials varied significantly among the 4 treatment systems (Figure 4), with a higher value (+320 ± 20 mV) found in the non‐bioaugmented CW planted with *J. effusus* than in the unplanted CW (−168 ± 22 mV). As expected, the oxic system works more efficiently than the anoxic one. Aerobic ibuprofen removal is known to be faster than anaerobic removal 40.

It is important to note the highly different removal efficiencies found in the three non‐bioaugmented wetland setups (CW‐J, CW‐P, and CW‐U) and their direct relationship with the redox values. Ávila et al. 41 showed a direct correlation between higher redox potentials in CWs and ibuprofen removal. It is also important to note that immediately after the addition of bioaugmented gravel in CW‐B, the redox potential dropped from 307 to 234 mV and then stabilized after the first month at an average of 240 ± 6 mV until the end of the experiment. This redox potential change clearly shows how the addition of ibuprofen as a new carbon source followed by its microbial degradation, increased the oxygen consumption in the CW. Oxic (redox potential > 100 mV) conditions favor the biodegradation of organic micropollutants through the promotion of biochemical reactions 42. Ibuprofen, naproxen, salicylic acid, and caffeine are all better removed under oxic conditions 3.

The average pH of the artificial wastewater in the inflow was 7.5 (data not shown). The pH of the outflow from all planted CWs decreased to a range of 6.0–6.6 as indicated in Figure 3. On the other hand, the pH values in the unplanted system remained close to the initial value (7.0–7.25) until the end of the experiment. The reason for this difference between the unplanted and the planted systems might be the formation of dissolved carbonic acid and carbon dioxide in water due to the degradation of organic compounds, like root exudates by aerobic organisms, resulting in pH reduction 43. Additionally, the tendency of plants to decrease the pH of soil is ascribed to H^+^ excretion in exchange for root uptake of cations in concert with root exudation of organic acids and release of carbon dioxide from the root respiration 44.

## 4 CONCLUDING REMARKS

Addition of gravel bioaugmented with an ibuprofen‐degrading bacterium, to newly started constructed wetlands could improve the removal efficiency of ibuprofen and also greatly reduce the time required for its stabilization. Plants were shown to play a vital role for microbial processes and their maintenance in constructed wetlands. This experiment showed that more than 80 days of CW ibuprofen treatment in newly started wetland systems could be spared with an appropriate bioaugmentation technique and sensible selection of plant species.

Yet, not all constructed wetlands systems did reach 100% removal efficiency during this experiment. This might be explained by the heterogeneity of the constructed wetland system, in vertical and horizontal way as shown by Imfeld et al. 2009 7. As discussed in Section 3.1, ibuprofen removal by bacteria is highly dependent on oxygenation of the water by plants. The roots of neither *J. effusus* nor *P. arundinacea* did cover the entire gravel bed, leaving unaerated areas, mainly at the bottom of the constructed wetland. Another explanation for the incomplete removal could be the retention time of the system, which in our experiment was 2.5 days. A longer retention time might increase the efficiency, due to longer interaction time between ibuprofen and the bacterial communities responsible for its removal. Therefore, further experiments are necessary in order to find the optimal ibuprofen concentration/hydraulic retention time for optimal targeted compound degradation.

## CONFLICT OF INTEREST

The authors have declared no conflict of interest.
